# Improved outcomes for patients with autonomic movement disorders originally evaluated for psychogenic non-epileptiform spells

**DOI:** 10.3389/fneur.2026.1839185

**Published:** 2026-09-07

**Authors:** Alyssa Khoo, Madelynn Paul, Nora Rady, Matthew Sunda, Jacob Brik, Okeanis Vaou, Anna Hohler

**Affiliations:** 1 Boston Medical Center Brighton, Boston, MA, United States; 2 Tufts University School of Medicine, Boston, MA, United States; 3 Boston University, Boston, MA, United States; 4 Department of Neurology, University of Texas San Antonio, San Antonio, TX, United States

**Keywords:** autonomic dysfunction, autonomic movement disorders, functional neurological disorder, misdiagnosis, psychogenic non-epileptiform spells

## Abstract

**Background:**

Patients experiencing autonomic storms related to dysautonomia may present with abnormal movements, including convulsions, tremors, and myoclonus. Due to these nonspecific symptoms, they are often misdiagnosed with functional neurological disorders, including psychogenic non-epileptic spells (PNES), resulting in delayed or inappropriate treatment.

**Methods:**

This cohort study included 17 adult patients who presented to St. Elizabeth’s Medical Center’s emergency department with abnormal seizure-like activity. Inclusion criteria required non-epileptiform movements, confirmed by negative rEEG or cEEG testing, followed by a misdiagnosis of a functional neurological disorder, and referral to Neurology. Demographic and clinical information was collected from the electronic health record to describe the clinical pattern and treatment responses in patients with AMD initially diagnosed with PNES.

**Results:**

Among the 17 patients (male [*n* = 6], female [*n* = 11]), with a mean baseline age of 52 and a median age of 64, the most common movement phenotypes were tremor (*n* = 6), and myoclonus (*n* = 6). Other neurological manifestations were obtundation (*n* = 10) and presyncope (*n* = 6). All participants showed features of orthostatic hypotension, indicating possible dysautonomia. 14 of 17 patients were started on at least one medication for symptom management, including fludrocortisone, pyridostigmine, midodrine, and propranolol, with 8 of 17 on combination therapy. 10 of 17 patients who underwent pharmacological interventions reported improvement, including a decrease or resolution of abnormal movements, reduction of autonomic dysfunction symptoms, and fewer readmissions.

**Conclusion:**

Patients presenting with abnormal seizure-like movements confirmed as non-epileptic after further evaluation should be assessed for autonomic dysfunction. Treatment targeting orthostatic hypotension may help alleviate these abnormal movements.

## Introduction

The autonomic nervous system is comprised of the sympathetic, parasympathetic, and enteric nervous systems and plays a paramount role in the maintenance of homeostasis in the body (1). Dysfunction of the autonomic nervous system, also known as dysautonomia, can manifest itself as a variety of conditions and a wide range of symptomology such as syncope, presyncope, orthostatic intolerance, palpitations, or fatigue, due to the significant role of the autonomic nervous system throughout the body (2). Dysautonomia can be difficult to diagnose and is often misdiagnosed as a functional neurological disorder due to the overlapping features of episodic symptoms, paroxysmal events, and changes in the level of consciousness, along with normal structural imaging (3).

Functional neurological disorders are conditions characterized by altered brain network functioning rather than structural neurological abnormalities (4–6). Functional neurological disorders in the clinical setting often yield unremarkable neurological workups and are often a diagnosis of exclusion. Due to similarities in presentation, syndromes of autonomic dysfunction such as postural orthostatic tachycardia (POTS) are often misdiagnosed as functional neurological disorders (3). For instance, myoclonus, tremors, convulsions, and abnormal movements, common presenting symptoms in POTS, are also present in functional neurologic disorders (3). In addition, both POTS and functional neurological disorders are often comorbid with psychological disorders (3, 6–8).

A potential mechanism contributing to such abnormal movements is transient cerebral hypoperfusion caused by impaired autonomic cardiovascular regulation. POTS can impair maintenance of adequate cerebral blood flow during orthostatic stress or positional changes (9). This lack of adequate cerebral blood flow causes cerebral hypoxia and hypoperfusion which has been shown to produce involuntary motor instances such as myoclonus, tonic or dystonic movements, automatisms, and convulsive activity during syncopal episodes (10). As stated earlier, these manifestations could be improperly diagnosed as being due to functional neurologic disorders despite arising from an underlying physiological process. Recognizing cerebral hypoperfusion as a mechanism causing abnormal movements in dysautonomia may be helpful in explaining the overlap in presentation between autonomic dysfunction and FND and reduce the risk of misdiagnosis. With this information, we propose a new definition of Autonomic Movement Disorder, which represents alteration in cognitive response with or without tremor, myoclonus, or dystonia related to a disruption of perfusion to the cortex as can be seen with orthostatic hypotension or postural tachycardia syndrome. This new definition is to be built upon with further research exploring the mechanisms and effects of Autonomic Movement Disorders.

The burden patients with dysautonomia experience when they are misdiagnosed with functional neurologic disorder is all-encompassing. Workup for neurological disorders in general tends to be costly due to all the necessary tests, imaging, and labs. Workup for functional neurological disorder has been observed to cost as much as and often more than other chronic neurological conditions such as multiple sclerosis and epilepsy (6). Misdiagnosis prevents patients from receiving the proper treatment that they need to mitigate their symptoms. In addition, literature suggests that once patients are provided with a diagnosis of Functional Neurological Disorder (FND), it makes it difficult for them to acquire further diagnostic testing or therapeutic care because additional symptoms are often attributed to a psychiatric diagnosis (11). Ultimately, this contributes to a significant decrease in quality of life, which occurs not only in the outpatient setting but often also leads to hospital admissions and re-admissions due to untreated symptoms.

While our study focuses primarily on mitigating abnormal movement symptoms, the medical management that comes with a proper diagnosis of an autonomic movement disorder can help address other symptoms of autonomic dysfunction long-term, thus reducing the need for hospital readmission. Medications used for the treatment of autonomic disorders include fludrocortisone, droxidopa, pyridostigmine, midodrine, beta-blockers, and combinations of these therapies, which alleviate symptoms of dysautonomia such as syncope, fatigue, palpitations, tachycardia, and orthostatic hypotension. They also help to reduce the movement component (tremors, myoclonus, and convulsions) (2, 12). By identifying patients initially diagnosed with PNES that may also have an underlying autonomic dysfunction, we can ensure that they receive the proper treatment, not only reducing episodes of abnormal movement, but also reducing the need for readmission to the hospital. This study aims to describe the clinical pattern and treatment responses in patients with AMD initially diagnosed with PNES in order to help identify misdiagnosed patients and improve future clinical outcomes.

## Methods

This retrospective cohort study analyzed clinical data from 17 adult patients who presented to the emergency department (ED) at St. Elizabeth’s Medical Center between January 1, 2022, and April 1, 2025, with abnormal seizure-like movements. The study received exemption from the Institutional Review Board (IRB) at St. Elizabeth’s Medical Center, and all procedures adhered to ethical standards for research involving human subjects.

Inclusion criteria required movements captured during EEG recording without corresponding epileptiform activity, confirmed by negative routine electroencephalography (rEEG) or continuous electroencephalography (cEEG) testing. Only those who subsequently received a clinical diagnosis of PNES made by an epileptologist were included. In addition, these patients had to be referred to Neurology outpatient services for further evaluation and management following their ED visit. Patients were excluded from the study if they had confirmed epileptic activity on EEG, structural brain abnormalities on neuroimaging, or insufficient documentation within the electronic health record (EHR).

Clinical and demographic data were extracted from the hospital’s electronic health record (EHR) system. Collected information included patient age, sex, past medical history, admission dates, descriptions of seizure-like symptoms at each admission, initial clinical impressions, and differential diagnoses considered prior to the involvement of the Neurology service and consideration of an autonomic component. Additional data included results of EEG testing, orthostatic vital signs, final diagnoses, medications initiated, treatment efficacy, discharge dates, and follow-up information when available. Orthostatic blood pressure was measured by having the patient lie down for 5 min prior to having an initial blood pressure and pulse rate measured. The patient was then asked to stand, and the blood pressure and pulse rate were measured after standing at 1 min and 3 min.

Given the limited sample size and the descriptive objectives of the study, data analysis was primarily qualitative. Descriptive statistics, including means, medians, ranges, and frequencies, were used to summarize patient characteristics and clinical variables. No statistical analyses were performed due to the exploratory nature of the investigation and low statistical power.

## Results

**Figure 1:**
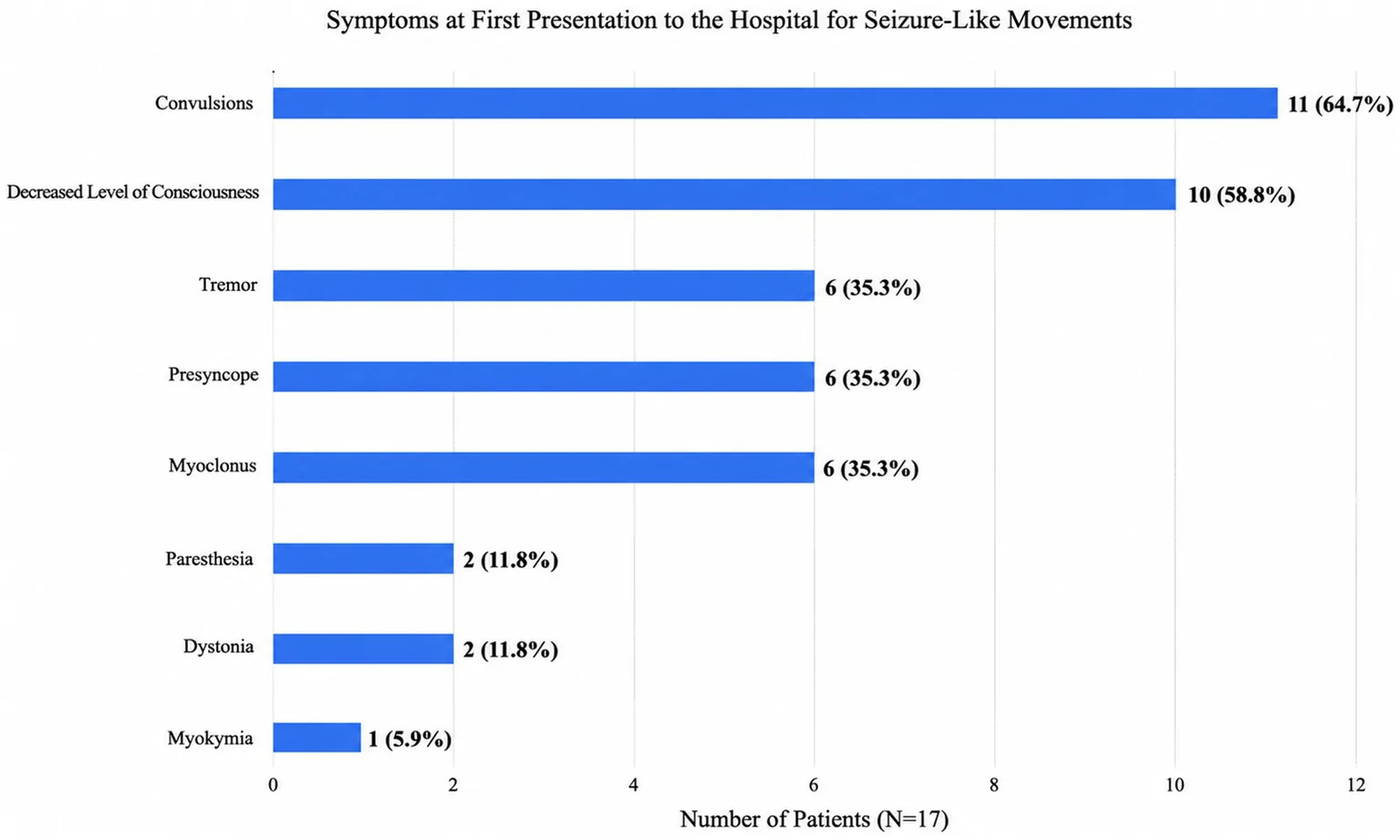
Clinical symptoms reported by patients at initial presentation for abnormal movements who were subsequently evaluated for dysautonomia. Symptoms include both motor and non-motor manifestations.

Out of the 17 patients (male [*n* = 6], female [*n* = 11]), with a mean baseline age of 52.8 and a median baseline age of 64, patients manifested a spectrum of abnormal seizure-like motor symptoms, including bilateral lower limb shaking, generalized tonic–clonic activity, orofacial frothing, rapid bilateral eyelid fluttering, focal muscle spasms, convulsive episodes, and intermittent limb myoclonus. The most common movement phenotypes were tremor (*n* = 6), and myoclonus (*n* = 6) (Figure 1). Other neurological manifestations were obtundation (*n* = 10) and presyncope, defined as patient-reported or observed near-fainting episode, often accompanied by lightheadedness, weakness, and visual changes lasting for a few seconds to several minutes (*n* = 6).

All of the participants showed features of either orthostatic hypotension or postural tachycardia when the blood pressure and heart rate were initially performed using standard testing at the bedside, indicating possible dysautonomia. This diagnosis of orthostatic hypotension was later confirmed on tilt table testing. Orthostatic hypotension was defined as a drop in systolic blood pressure of ≥ 20 mm Hg or a diastolic blood of ≥10 mm Hg within 3 min of standing or during a head-up tilt of at least 60 degrees. A positive result for a POTS diagnosis is defined by a heart rate increase of ≥30 beats per minute within 10 min of standing or tilting, without a significant drop in blood pressure (13). Using this data and analysis of the movements, a diagnosis of AMD was made by a movement disorder specialist. All of these patients received conservative therapies with fluids, electrolytes, and compression stockings. It was found that 14 of the patients were started on at least one medication to manage their symptoms, including fludrocortisone, pyridostigmine, midodrine, and beta-blockers, with 8 on combination therapy. 10 of the patients who underwent pharmacological interventions reported improvement, including a decrease or complete resolution of abnormal movements and fewer readmissions (Table 1).

**Table 1:** Summary of patients evaluated for suspected seizures, categorized by outcome.

Case	Age/Sex	Event semiology leading to consideration of epilepsy/seizure	Clinical evaluation	Outcome
1	78/M	Recurrent bilateral and unilateral limb shaking	rEEG and cEEG normal; positive orthostatic vitals	Improvement of motor symptoms and orthostatic vitals with medication
2	34/F	Episodes of shaking with reduced responsiveness	rEEG normal; positive orthostatic vitals	Improvement of motor symptoms and orthostatic vitals with medication
3	21/F	Repetitive shaking of the upper and lower extremities with oral frothing	cEEG normal; positive orthostatic vitals	Improvement of motor symptoms and orthostatic vitals with medication
4	21/F	Brief focal motor event with preserved awareness, followed by bilateral limb twitching and eyelid fluttering	rEEG and cEEG normal; positive orthostatic vitals	Improvement of motor symptoms and orthostatic vitals with medication
5	24/F	Brief bilateral hand shaking with staring and unresponsiveness, without vocalization	rEEG and cEEG normal; positive orthostatic vitals	Improvement of motor symptoms and orthostatic vitals with medication
6	71/F	Brief episode of blank stare and unresponsiveness with unilateral right-sided twitching and leg involvement; later developed transient left facial droop and language abnormality	cEEG normal; positive orthostatic vitals	Improvement of motor symptoms and orthostatic vitals with medication
7	80/M	Involuntary jaw opening with inability to close the mouth, accompanied by lateral head bobbing	rEEG normal; positive orthostatic vitals	Improvement of motor symptoms and orthostatic vitals with medication
8	75/M	Frequent presyncope and recurrent convulsive syncope with post-event confusion	rEEG showed evidence of mild encephalopathy with no epileptiform or ictal features; positive orthostatic vitals	Improvement of motor symptoms and orthostatic vitals with medication
9	64/F	Tonic–clonic episodes with transient unresponsiveness and rapid return to baseline, without significant postictal confusion.	cEEG normal; positive orthostatic vitals	Improvement of motor symptoms and orthostatic vitals with medication
10	19/F	Recurrent bilateral leg shaking with distal paresthesias	rEEG normal; positive orthostatic vitals; previously diagnosed with POTS; symptoms include repeated episodes of dizziness, fatigue, and frequent falls	Improvement of motor symptoms and orthostatic vitals with medication
11	71/F	Fluctuating mental status with bilateral arm myoclonic-appearing movements	cEEG showed unevenly modulated background with no epileptiform or ictal features; positive orthostatic vitals	Improvement of motor symptoms and orthostatic vitals with medication
12	35/F	Episode of lower-extremity abnormal movements with whole-body contractions and facial grimacing	rEEG and cEEG normal; positive orthostatic vitals	Started on medication; lost to follow-up; treatment response unknown.
13	89/F	Recurrent near-syncope with lightheadedness, diaphoresis, and generalized weakness	cEEG normal; positive orthostatic vitals	Started on medication; lost to follow-up; treatment response unknown.
14	35/M	Intermittent limb shaking with loss of consciousness	rEEG normal; positive orthostatic vitals	Lost to follow-up; treatment response unknown.
15	28/M	Transient unresponsiveness with blank stare, preceded by lightheadedness	rEEG normal; positive orthostatic vitals	Lost to follow-up; treatment response unknown.
16	73/F	Presyncopal dizziness with transient unresponsiveness and brief confusion	rEEG showed slower than typical background when in wakefulness with no epileptiform or ictal features; positive orthostatic vitals	Lost to follow-up; treatment response unknown.
17	79/M	Apparent postictal confusion/disorientation after LOC while driving	rEEG normal; positive orthostatic vitals	Lost to follow-up; treatment response unknown.

Clinical evaluation describes EEG testing (routine and continuous EEG) and autonomic assessment with orthostatic vitals; outcome reports the response to intervention.

## Discussion

Recognizing symptoms of autonomic dysfunction during a patient’s initial emergency department visit can help ensure appropriate treatment, thereby reducing the risk of premature discharge and subsequent hospital readmissions. One trend observed in our study was that many of the patients had presented multiple times to the emergency department before being referred for neurology evaluation. Some of these patients from our cohort presented up to seven times to the emergency department before they were provided with a neurology referral. This presents an issue for the patient as they ultimately do not receive treatment that alleviates their symptoms, creating a cycle in which they re-present back to the emergency department and accrue a financial burden until they ultimately receive some sort of solution or relief of their symptoms. The observation that patients are presenting multiple times to the emergency department before they are referred to outpatient neurology suggests a significant gap in patient care that we hope can be addressed through recognition of symptoms that have been addressed in this study.

**Figure 2:**
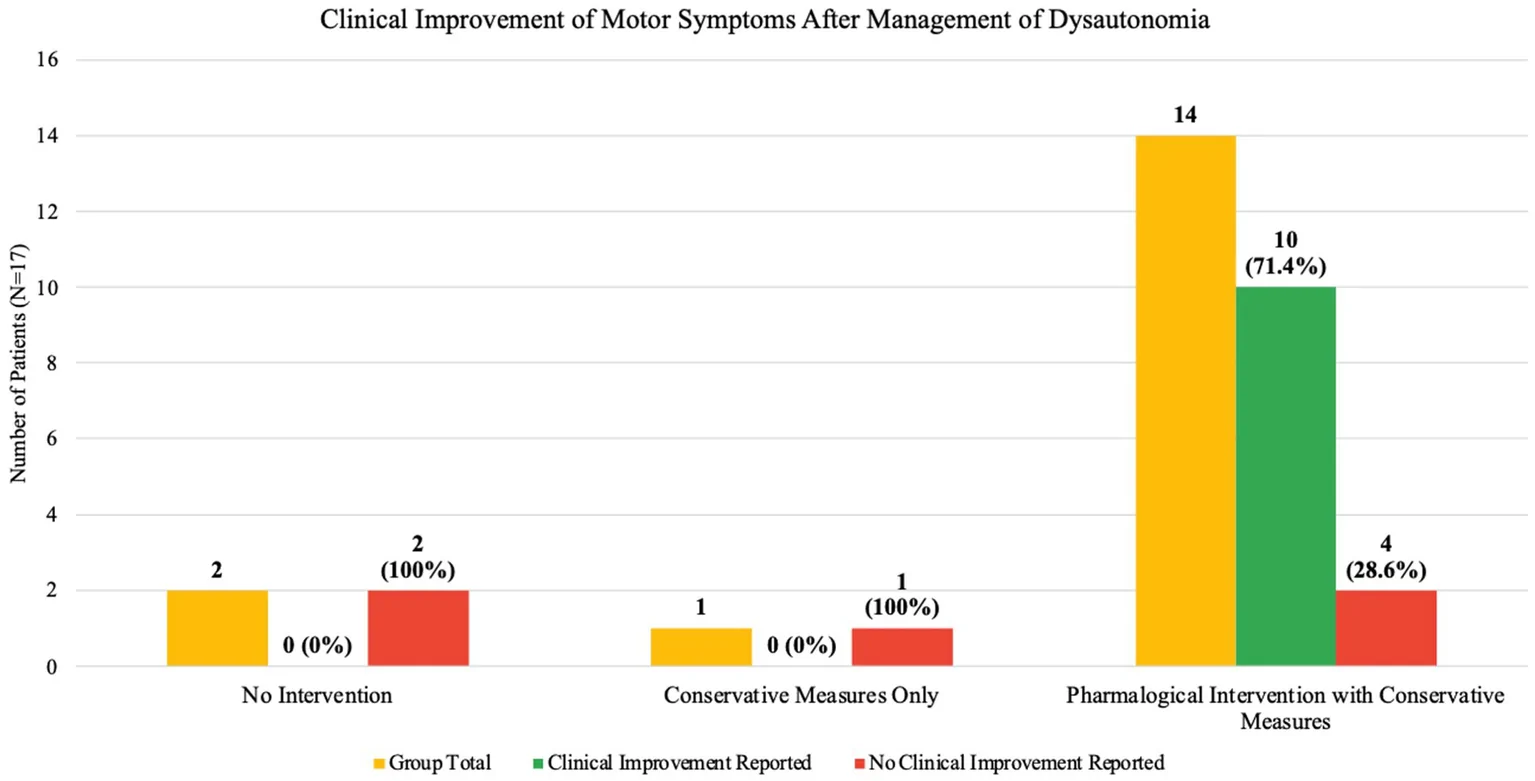
Clinical improvement of abnormal movements following management of dysautonomia. Patients were categorized into three groups: no intervention, conservative measures (e.g., electrolyte supplementation, compression stockings, and increased salt intake), and conservative measures with pharmacologic treatment. Percentages indicate the proportion of patients in each group who experienced a reduction or complete resolution of abnormal movements.

One of the most significant benefits that comes with patients receiving the proper diagnosis of dysautonomia is the ability to access treatments that otherwise would not be offered to them with a diagnosis of PNES. When a patient is misdiagnosed as having PNES, it delays treatment or may restrict treatment entirely. In our study, 14 patients were started on at least one pharmacological treatment mentioned previously, and 8 patients started on a combination therapy including multiple therapeutics to manage their symptoms once they were properly diagnosed. In total, 10 of these patients who underwent pharmacological interventions showed improvements, both self-reported and clinically assessed by the Movement Disorder Specialist, including a decrease in or complete resolution of abnormal movements, and fewer hospital readmissions when assessed at both the 3-month and 6-month mark (Figure 2). Upon treatment, we also observed patients with improvements in orthostatic vitals, with one patient stating that receiving proper treatment and seeing clinical improvements “felt like a weight being lifted”. In addition to these pharmacological therapies, additional therapies were offered to patients, including compression stockings, IV fluids, electrolytes, and vitamins.

The patients in our cohort presented with a wide variety of symptoms, prompting clinicians to work up for FND. These symptoms included tremor, myoclonus, obtundation, presyncope, and orthostatic hypotension. The lack of recognition of symptoms of dysautonomia, along with the wide overlap with FND, contributes to the continued misdiagnosis of these conditions, leading to unnecessary burdens being applied to patients. Although patients in our cohort exhibited a variety of dysautonomic and abnormal movement symptoms, it should be noted that all of the patients in our cohort who presented to the ED exhibited orthostasis. This could signify a differentiating symptom to watch out for and can point toward a diagnosis of dysautonomia rather than FND. This suggests that in the presence of a negative EEG result and symptoms resembling a seizure, performing a tilt-table test and checking orthostatic vitals prior to FND diagnosis can prove to be very useful with respect to guiding the clinician in the selection of further testing and differential diagnosis.

It is important to acknowledge that autonomic dysfunction and functional neurologic disorder are not necessarily mutually exclusive diagnoses. A recent study addresses a possible overlap between the two conditions, though the exact mechanism is unknown (14). The current hypothesis implicates shared neutral circuitry involving the limbic system and salience networks, which are involved in autonomic regulation and symptom perception. Another study had reported that patients with FND exhibit altered autonomic function, including higher resting heart rates and variability compared to healthy controls (15). Additionally, patients with autonomic dysfunction have high rates of psychiatric comorbidities, including anxiety, depression, and PTSD, further supporting the possibility of shared pathophysiologic pathways (16, 17). While the primary goal of this study is not to dispute the possibility of autonomic dysfunction and FNDs overlapping, our findings highlight the potential for a treatable autonomic component contributing to a patients’ seizure-like movements, when they are proven to be non-epileptiform. These findings support consideration of autonomic evaluation, including formal autonomic testing, as part of the diagnostic workup for patients presenting with unexplained seizure-like movements.

There are several limitations to our study. First, our study examined a small sample size of patients presenting a single site, and it would be beneficial to perform the study with a larger sample size from multiple sites, in order to examine whether orthostasis at presentation remains a key differentiating factor between PNES and AMD. Another limitation of this study is the possibility of selection bias due to only studying the population that was referred to Neurology and tilt table testing. Despite these limitations, our study shows the importance of further work up for patients who present with non-epileptiform seizure-like movements due to the possibility of AMD.

## Conclusion

Patients who present with abnormal seizure-like movements that are shown not to be epileptic after further workup, should be evaluated for autonomic dysfunction. Medications that manage orthostatic hypotension may help to resolve abnormal movements. Treatment is effective for this cohort of patients and has been observed to improve quality of life and avoid emergency department re-presentation and hospital re-admission. It is important to work up patients for orthostatic testing and signs of autonomic dysfunction when other neurological testing is negative and there is a suspected diagnosis of PNES and other functional neurological disorders.

## Data Availability

The raw data supporting the conclusions of this article will be made available by the authors, without undue reservation.
